# A Methodological Case Study for Integrating Virtual Reality and 3D Photogrammetry: A Pilot Digital Platform for Exploring Mangrove Environments

**DOI:** 10.1002/ece3.74410

**Published:** 2026-09-28

**Authors:** Terence Pun Tung Ng, Kenny Zhi Hao Li, Cheryl Wood Yee Shum, Kian Ka Ki Tam, Yiu Man Chan, Flora Siu Yan Mok, David Tai Wai Lau

**Affiliations:** ^1^ Agriculture, Fisheries and Conservation Department The Government of the Hong Kong Special Administrative Region Hong Kong Hong Kong Special Administration Region China; ^2^ Shiu‐Ying Hu Herbarium, School of Life Sciences The Chinese University of Hong Kong Hong Kong Hong Kong Special Administration Region China

**Keywords:** Mangroves, multimodal platform, photogrammetry, Ramsar Site, virtual reality

## Abstract

Mangroves are ecologically vital ecosystems that present unique logistical challenges for field‐based environmental education and research due to seasonal tidal fluctuations, difficult muddy terrain, and visitor safety considerations. Balancing sustainable conservation with accessible site exploration requires innovative approaches. To address these constraints, we developed a pilot multimodal platform to demonstrate the technical feasibility of integrating virtual reality (VR) and 3D photogrammetry as core methodological components for environmental representation. This interactive platform constructs virtual walking trails across two ecologically significant mangrove stands in Hong Kong, one within a Ramsar Site and another within a Site of Special Scientific Interest (SSSI). These sites serve as methodological testbeds to showcase how immersive digital environments can realistically represent complex, restricted terrains without geographical or temporal limitations. The virtual trails incorporate high‐resolution 3D models of representative mangrove flora and fauna (e.g., plants, snails and crabs), allowing users to examine intricate morphological details. Supplementary high‐resolution visual media of species and habitats are embedded to construct a comprehensive multimodal environment. As a methodological case study, this work establishes the practical workflow for combining VR and 3D photogrammetry. The resulting pilot platform now awaits future empirical testing to evaluate its potential efficacy and value for environmental education in sensitive ecosystems.

## Graphical abstract

This study presents a methodological prototype that demonstrates the potential of integrating virtual reality and 3D photogrammetry to address the logistical and safety challenges inherent in exploration within sensitive habitats, with particular focus on mangrove ecosystems. By showcasing how immersive digital environments can realistically represent complex, access‐restricted terrains free from geographical or temporal constraints, this work establishes a practical workflow for combining VR and 3D photogrammetry.

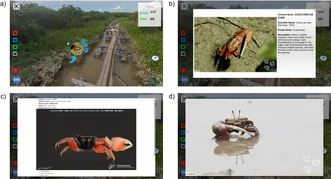

## 1 Introduction

### 1.1 Challenges for Field‐Based Learning in Mangroves

Mangroves are valuable habitats for environmental education and ecotourism because they allow visitors to observe rich biodiversity, ecological interactions, and coastal processes in situ (Tagulao et al. 2022; Verawati and Idrus 2023). However, their educational use is inherently difficult. Educational access is often constrained by conservation requirements, since many mangrove areas are ecologically sensitive and subject to restricted entry or closely managed visitation (Dabalà et al. 2023). Physical access to mangroves is further constrained by tidal variability, unstable muddy substrates, and weather‐related safety risks (Tam and Wong 2000), all of which limit the feasibility, frequency, and inclusiveness of field‐based learning.

Current educational approaches do not fully resolve these limitations. Field visits to mangroves can provide valuable first‐hand experience, but they are logistically demanding, seasonally constrained, and not always compatible with site protection (Ross 2006; Cárdenas et al. 2017). At the same time, conventional classroom materials, such as photos, videos, and text‐based resources, can introduce mangrove ecosystems, yet they offer only limited spatial immersion and do not adequately replicate the experience of exploring habitat structure or examining organisms in detail (Ou et al. 2021). As a result, there remains a methodological gap between the need to protect mangrove ecosystems and the need to make them accessible for education. Fortunately, modern technology offers a solution to bridge the gap between conservation and education of such important ecosystems by serving as a virtual complement to physical tours (Gambin et al. 2021; Singh 2025).

### 1.2 Potential for the Application of VR and 3D Photogrammetry in Environmental Education

VR and 3D photogrammetry are relatively new tools that have gained popularity in education (Kamińska et al. 2019; Chytas et al. 2024). VR can create immersive 3D environments that resemble real‐world settings. For example, Google Expedition, a VR platform designed for educational institutions, enables teachers to bring students on virtual reality trips to places like coastlines, museums, and other outdoor sites (Parmaxi et al. 2017; Cardullo and Wang 2021). On the other hand, 3D photogrammetry enables experiential teaching and learning in disciplines where physical access is challenging (Ohgashi et al. 1996; Burk and Johnson 2019). For example, authentic 3D digital models created using photogrammetry have effectively addressed the lack of cadaveric specimens in anatomy education (Petriceks et al. 2018). Recent studies show that high‐resolution 3D models generated through photogrammetric techniques can identify unique features of small plant parts or even tree trunks (Wang et al. 2022, 2024), suggesting strong potential for ecological and environmental education. Nevertheless, the combined use of VR and 3D photogrammetry in environmental education remains limited.

### 1.3 A Methodological Case Study

This research addresses the methodological gap between the need to protect mangrove ecosystems and the need to make them accessible for exploration by developing a pilot multimodal digital platform that integrates VR and 3D photogrammetry. Using Mai Po and Ting Kok mangroves in Hong Kong as demonstration sites, the study aims to show how immersive virtual trails and high‐resolution 3D models can provide an alternative and complementary mode of mangrove exploration. The objective is not to present a detailed ecological comparison of the two sites, but to illustrate the methodological potential of this digital approach for environmental exploration in ecosystems where direct access is constrained. Particularly for Mai Po, tours are available to students and the public under an entry quota system (AFCD 2026a). In addition, although mangroves in Hong Kong are excellent outdoor classrooms for field trips and citizen science projects, their accessibility and safety are greatly influenced by seasonal tidal and weather patterns. For example, low tides that favor close‐up access to mangroves often occur at night or dawn during the dry, cold season (November to February), while heavy rains and typhoons are common during the wet, hot season (June to September; Hong Kong Observatory 2026). Digital platforms can, therefore, potentially serve as valuable supplements to field‐based learning in local intertidal habitats. Additionally, they offer inclusive alternatives for students with disabilities who may find the unstable mangrove substrate physically challenging.

## 2 Methods

### 2.1 Study Sites

The pilot digital platform developed in this study features two important mangrove stands in Hong Kong, Mai Po and Ting Kok, both dominated by *Kandelia obovata*. These two sites were selected because they represent two major and popular mangrove stands in Hong Kong, located in the western and eastern waters, respectively.

The Mai Po mangrove, the largest local mangrove stand, together with the surrounding *gei wais* (traditional shrimp ponds), fishponds, mudflats and reedbeds, forms essential parts of the Mai Po Inner Deep Bay Ramsar Site (about 1500 ha), recognized as a Wetland of International Importance under the Ramsar Convention since 1995 (AFCD 2026a). This Ramsar Site serves as a crucial staging and wintering ground for migratory waterbirds along the East Asian–Australasian Flyway. Approximately 50,000–60,000 migratory waterbirds, including several globally threatened species such as Black‐faced Spoonbill, Saunders's Gull and Nordmann's Greenshank, roost in the area during winter (WWF‐HK 2024). Although the World Wide Fund for Nature Hong Kong (WWF‐HK), with support from the Agriculture, Fisheries and Conservation Department (AFCD) of the Hong Kong Special Administrative Region (SAR) Government, provides guided tours in the Mai Po area, the mangrove habitats are largely located within zones where access is restricted year‐round to minimize disturbance to wildlife.

Another study site, Ting Kok mangrove, is easily accessible by public transport and features a firm and compact mudflat. Covering about 8.77 ha, it is Hong Kong's fourth‐largest mangrove stand and the largest hard‐substrate mangrove, supporting rich biodiversity (AFCD 2026b). Home to six of Hong Kong's eight true mangrove species and a rich diversity of flora and fauna, the site was designated a Site of Special Scientific Interest (SSSI) in 1985 because of its ecological significance (AFCD 2026b). Its accessibility makes it a popular destination for school field trips and research studies (Tong et al. 2006; AFCD 2026b).

### 2.2 Study Period and Checkpoints

A workflow diagram illustrating the methodological approach is presented in Figure 1, with detailed procedures described below.

**FIGURE 1:**
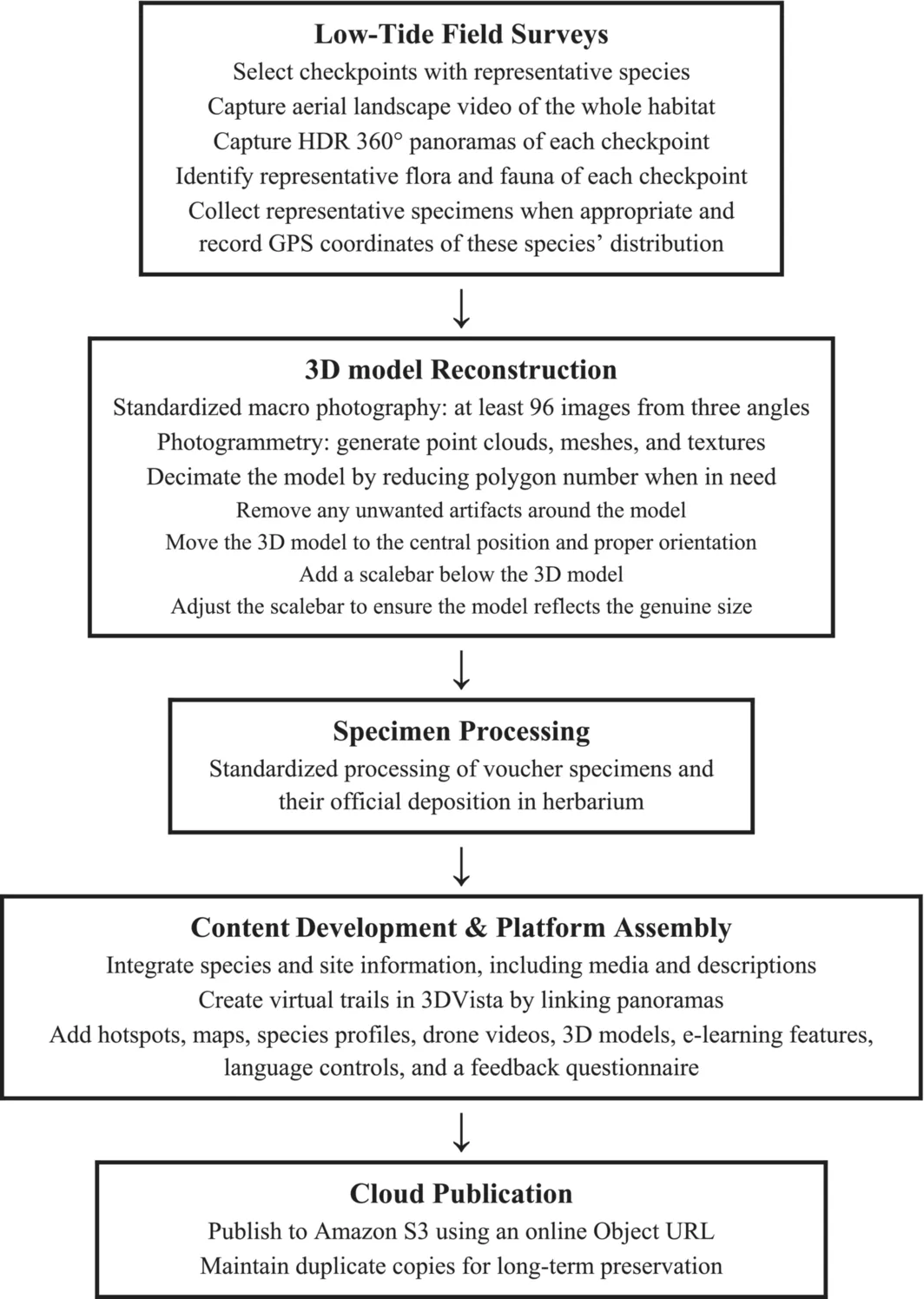
Workflow diagram illustrating the methodological approach.

To collect data and specimens for developing the digital platform, field trips were conducted during low tide periods (mostly below 1 m above Chart Datum). Three field trips were carried out along the floating boardwalk passing through the mangrove stand within the Ramsar Site on July 3, 2023, July 13, 2023, and December 11, 2023. Four trips were conducted to the eastern shore of the Ting Kok mangrove on June 5, 2023, June 30, 2023, December 11, 2023, and February 26, 2024. Five checkpoints were selected at each mangrove site (Table 1) for virtual trail construction based on ease of accessibility and presence of representative flora and fauna species. Here, “representative species” refers to those that are most abundant and/or readily observable.

**TABLE 1:** GPS coordinates of mangrove area checkpoints at Mai Po and Ting Kok mangroves.

Site	Checkpoint no.	Latitude	Longitude
Mai Po	Checkpoint 1	22.4948	114.0318
	Checkpoint 2	22.4953	114.0313
	Checkpoint 3	22.4951	114.0307
	Checkpoint 4	22.4951	114.0301
	Checkpoint 5	22.4953	114.0298
Ting Kok	Checkpoint 1	22.4677	114.2178
	Checkpoint 2	22.4683	114.2175
	Checkpoint 3	22.4687	114.2171
	Checkpoint 4	22.4690	114.2167
	Checkpoint 5	22.4694	114.2166

### 2.3 Panorama Photographing

At each checkpoint, an Insta360 X3 panorama camera was mounted on an extended selfie stick and handheld at an appropriate height of 2.5 m for photo shooting. The actual shooting height was adjusted depending on the surrounding environment or the key species to be highlighted. Images were captured using the high dynamic range (HDR) mode of the Insta360 X3 to enhance brightness and detail. After fieldwork, the panorama photos (INSP files) were exported and converted to JPG format using Insta360 Studio 2023.

### 2.4 Aerial Landscape Recording

An ANAFI drone equipped with a Parrot Skycontroller 3 was used to capture aerial footage of landscape features at both study sites. The drone angle and altitude were continuously adjusted to ensure comprehensive coverage of key features, including mangrove stands and adjacent habitats (e.g., *gei wais*, mudflats and reedbeds). To minimize disturbance to wildlife, the drone was operated at appropriate distances and avoided ecologically sensitive periods, such as the peak season for migratory winter birds.

### 2.5 Specimen Collection and Identification

Dedicated efforts were made to collect fresh dominant and representative mangrove flora and fauna species encountered at the checkpoints for 3D model reconstruction and as voucher specimens. No more than three samples per species were collected. Species identification was carried out by local taxonomic experts from the AFCD Wetland Specialist Working Group and the Shiu‐Ying Hu Herbarium of The Chinese University of Hong Kong. The GPS coordinates were recorded for each voucher specimen, along with key authenticating features such as flowers or fruits for plants. After standard processing, the specimens were deposited in the Shiu‐Ying Hu Herbarium for potential future research and educational use.

### 2.6 3D Model Reconstruction

Following Wang et al. (2022), 3D models of the sampled mangrove flora and fauna specimens were reconstructed. A Canon EOS R5 camera equipped with an EF 100 mm macro lens was used, with settings fixed at a shutter speed of 1/60s, aperture of f/16 and ISO between 100 and 200. Specimens were mounted on a rotating platform and illuminated by three Phottix M200R RGB lights positioned on the left, right and top to ensure uniform lighting. The lights were calibrated to a color temperature of 5600 K to mimic natural white light and capture authentic specimen colors. For each specimen, a minimum of 96 photos were taken, 32 each from the three vertical view angles (approximately +20° to +25°, 0°, and −20° to −25°), with the platform rotated by 1/32 of a full rotation after each shot. Complex material additionally used approximately +35° and −35° views. A customized marker bearing ruler was placed with the specimen in every capture so that the models have in‐scene reference for scale. The models were scaled to represent specimens as small as about 3 mm. All photos were imported into 3DF Zephyr Lite (version 7.5) for photogrammetric processing, including point cloud reconstruction, mesh generation and textured mesh generation. Most original models had more than one million faces (or polygons) from the generation, making it almost impossible to load on edge devices. Therefore, most models were decimated to around 200 k faces. The texture resolution was 8 k (8192 pixels in width and 8192 pixels in height). Efforts were made to correct the most common processing error, which was the camera alignment failure. Final refinements of the models were made using Blender software (version 3.6.0).

### 2.7 Construction of Virtual Trails

The media software 3DVista Virtual Tour (version 2024.0.11) was used to construct the two virtual trails consist of check points made from panorama photos, with interactive elements. Firstly, panorama photos were imported into 3DVista Virtual Tour, and the “Open Panorama” function was used to connect the panoramas. Interactive elements such as species information were added via the “Hotspot” function, while the “Open Info Window” function integrated photos and descriptive text. Videos and 3D models were embedded using the “Popup Web Frame” function, and site maps were customized within the “Floor Plans” interface. Checkpoints were marked using the “Hotspot” and “Radar” functions, with panoramas added to each checkpoint using the “Open Panorama” function. Under the digital module, the “Count to Score” function created a scoreboard to track the number of species users successfully explored along the virtual trails. The “Skin” element's “Show/hide” function enabled dynamic displays, including user guides, maps and species selections, and language switching. An introductory drone video was also incorporated using the “Hotspot” and “Popup Web Frame” functions. A questionnaire was incorporated at the final checkpoint of the virtual trails to collect feedback for evaluating user experience and learning outcomes in the future. Upon completion, the virtual trails were then published and exported to Amazon S3, with access provided via an online Object URL link. To ensure data preservation, backup protocols were implemented through data duplication, supporting the long‐term operation and sustainability of the digital platform.

## 3 Results

Two pilot virtual trails showcasing the biodiversity of the Mai Po and Ting Kok mangroves were developed as a methodological case study for a pilot multimodal digital platform, based on VR and 3D photogrammetry. All associated biodiversity data, along with the operating software, were stored on a customized Amazon S3 cloud server. The two virtual trails are accessible via the following links: https://cuhk‐static‐content.s3.ap‐southeast‐1.amazonaws.com/vr/Mai+Po+Inner+Deep+Bay/index.html and https://cuhk‐static‐content.s3.ap‐southeast‐1.amazonaws.com/vr/Ting+Kok+East+Shore/index.html. The trails are accessible on tested mobile and desktop devices. Additionally, users can explore the virtual trails using VR glasses for a more immersive experience. The questionnaire, which is also available at the final checkpoint of the trails, can be accessed at https://docs.google.com/forms/d/e/1FAIpQLSeXLH_FNPvp‐rjO‐_FduQXpvHV4‐KL5oNRQLGZ_xD1PcFVkYQ/viewform.

### 3.1 User Interface

The interface of both virtual trails includes an introductory video, accessible via the drone icon (Figure 2a), which provides background information and highlights the ecological significance of each study site. A range of interactive features is designed to help users explore and learn about the mangrove flora and fauna. Key interface elements include: species group selection buttons (Figure 2b) to display specific organism groups, user guideline (Figure 2c) offering clear instructions for navigation and interaction, language toggle button (Figure 1d) for switching between Chinese and English, interactive map thumbnail (Figure 2e) for navigation, scoreboard (Figure 2f) to track species discovered, navigation arrow (Figure 2g) to advance to the next checkpoint, interactive hotspots represented by magnifier icons (Figure 2h) for providing detailed species information, photos, videos, digitized specimens, and 3D models for species. The major mangrove organism groups covered include plants, crabs, mudskippers, snails, and waterbirds, offering a comprehensive exploration of local mangrove biodiversity.

**FIGURE 2:**
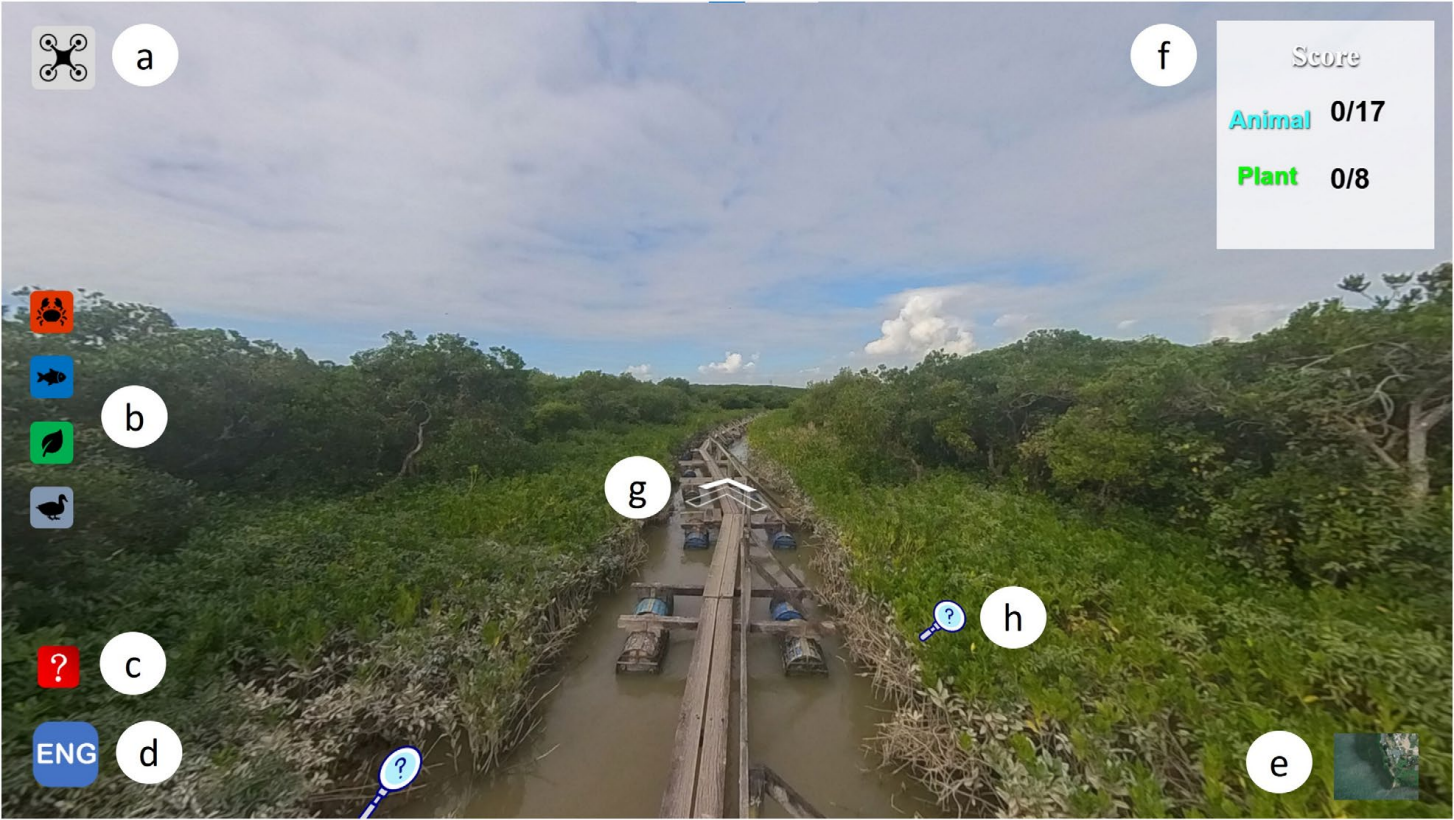
Screenshot of the virtual trail interface for the Mai Po mangrove. Key features include: (a) drone icon, (b) species group selection buttons, (c) user guideline, (d) language toggle button, (e) map thumbnail, (f) scoreboard, (g) navigation arrow for advancing to the next checkpoint, and (h) interactive hotspot.

### 3.2 Multimodal Platform and Biodiversity Inventory

A total of 14 and 19 representative flora and fauna species are featured along the Mai Po and Ting Kok mangrove virtual trails, respectively. Among these, 3D models were constructed for 4 species of plants, 9 species of crabs, and 9 species of snails. Each hotspot presents species‐specific content, including descriptions, high‐resolution photos, videos, 3D models, and/or digitized specimens. For example, the fiddler crab, *Tubuca arcuata*, found in the Mai Po mangrove, is presented with species information, a 3D model, and a video (Figure 3); the mangrove plant, *Excoecaria agallocha* , found in Ting Kok mangrove, is presented with species information, a digitized specimen, and a 3D model of its fruit (Figure 4). This multimodal approach enhances the educational value of the platform. The 3D models are of high enough resolution to distinguish not only between species (Wang et al. 2022) but also between sexes within the same species, as explained in Figure 5.

**FIGURE 3:**
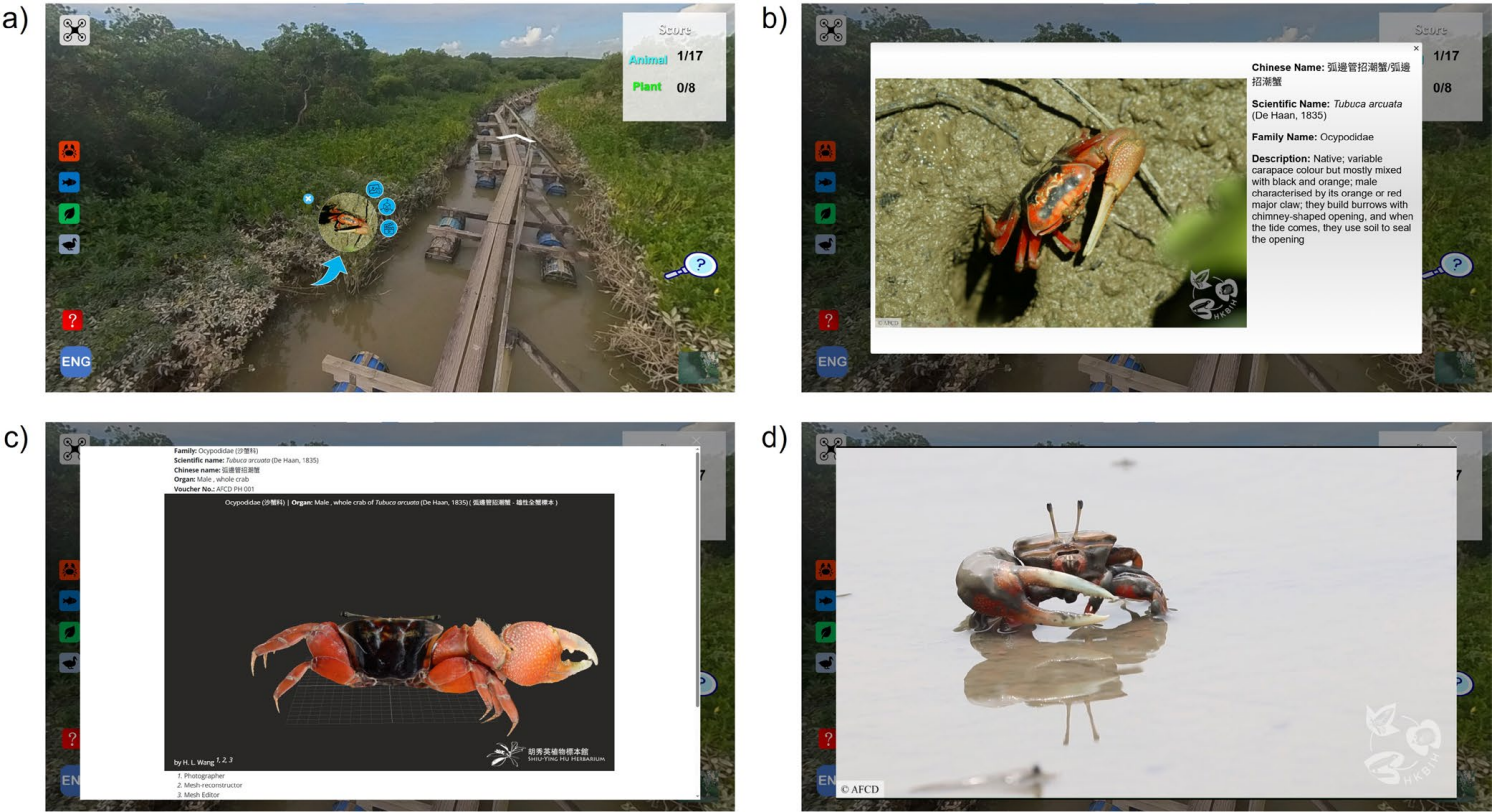
Example display of *Tubuca arcuata* on the digital platform. Key features include: (a) screenshot showing hotspot selection at a checkpoint along the Mai Po virtual trail, (b) species description panel, (c) high‐resolution 3D model of the species, and (d) embedded video showcasing species behavior.

**FIGURE 4:**
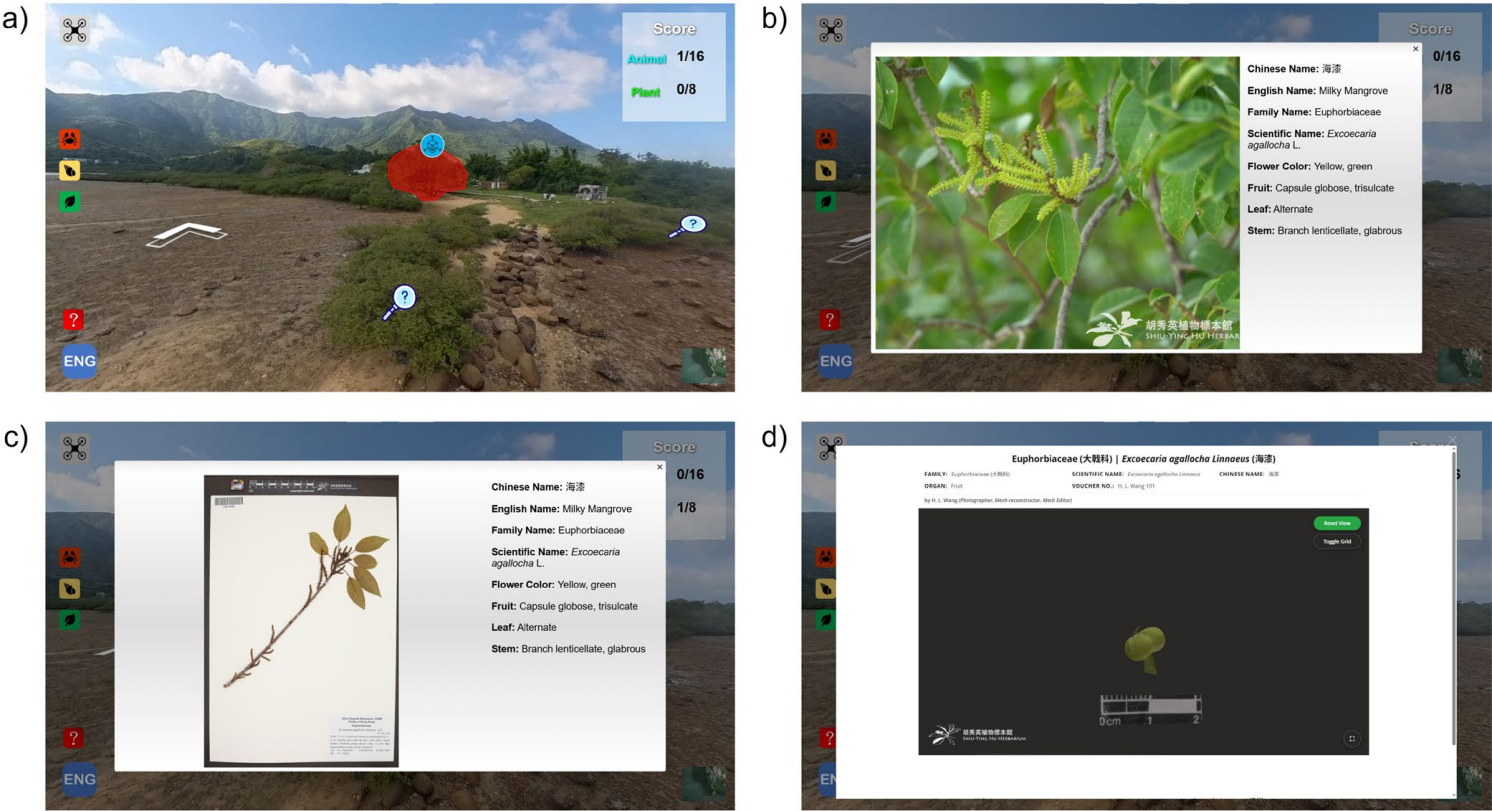
Example display of *Excoecaria agallocha* on the digital platform. Key features include: (a) screenshot showing hotspot selection at a checkpoint along the Ting Kok virtual trail, (b) species description panel, (c) digitized specimen of the species, and (d) high‐resolution 3D model of the plant's fruit.

**FIGURE 5:**
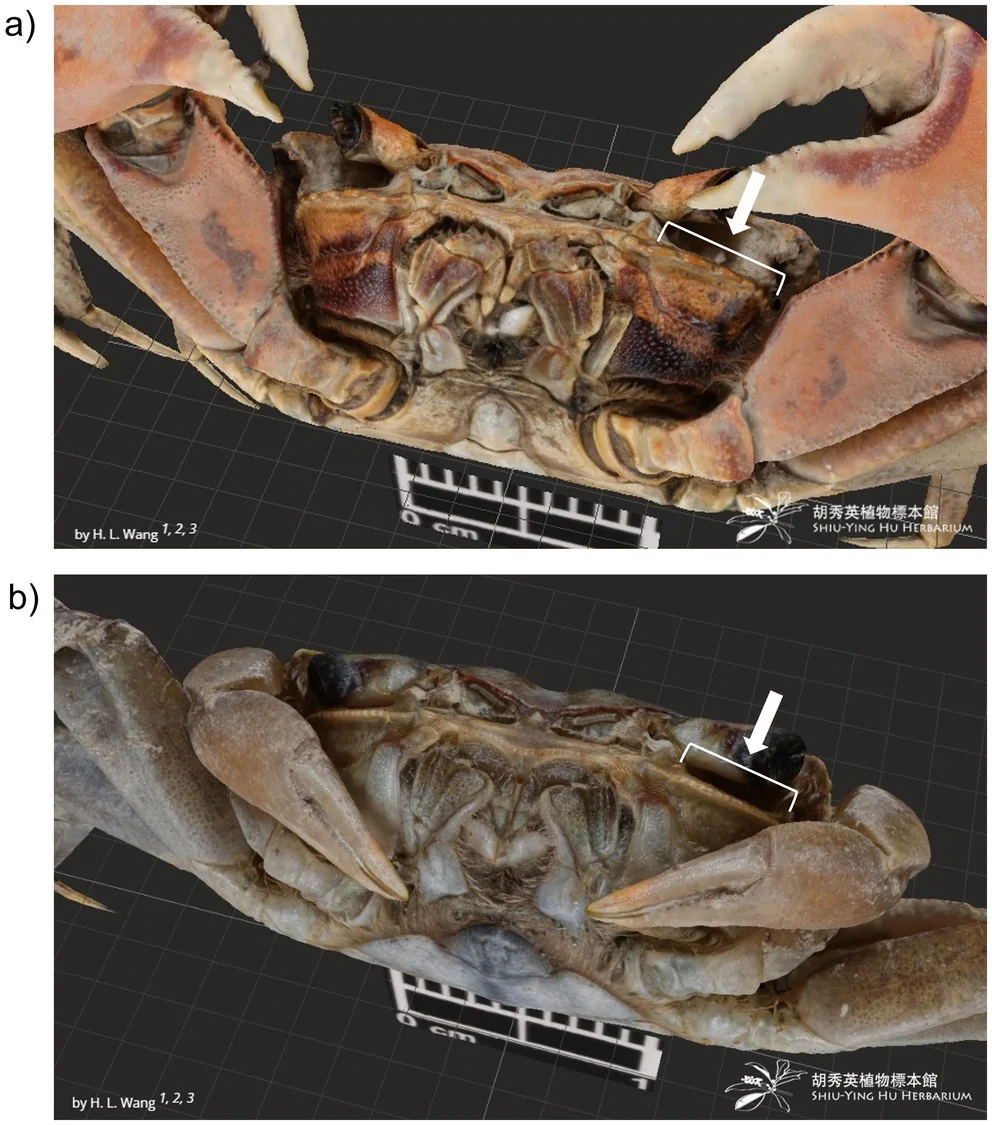
3D models of (a) male and (b) female of the mangrove crab, *Metaplax tredecim*. Apart from the typical trait for distinguishing between sexes of crabs (i.e., male crabs usually have narrow, triangular abdomens, whereas female crabs have wider, rounded abdomens), crab species often have other key identification traits between sexes, such as the infraorbital ridge in this species indicated by the white arrows. High resolution of this trait permits reliable identification of the two sexes. Infraorbital ridge of male *M. tredecim* has 13–20 tubercles, and the mesial tubercles are broad and then decrease in size, whereas that of female has 20–27 narrow tubercles of similar size (Shih et al. 2019).

All species‐specific content and digitized materials were validated, and voucher specimens of all species with 3D models have been permanently archived in the Shiu‐Ying Hu Herbarium. As a result, the biodiversity information and materials presented in both virtual trails are rigorously verified and preserved, forming a scientifically reliable inventory that can support further research and education.

## 4 Discussion

### 4.1 Virtual Technology for Sustainable Nature Appreciation

Traditional field‐based learning and ecotourism, while effective in fostering appreciation for nature, may negatively impact sensitive wildlife and, in extreme cases, lead to a decline in habitat quality (Blangy and Mehta 2006; De Zoysa 2022). However, the rapid advancement of computing and information technologies in the digital era presents new opportunities for immersive learning and virtual sightseeing within indoor environments (Rubio‐Tamayo et al. 2017). For example, VR tools enable users to explore ecologically sensitive areas, such as coral reefs, old‐growth forests, and endangered wildlife habitats, without physically entering the area (Vercelloni et al. 2018; Folgado‐Fernández et al. 2023). Using two ecologically significant mangrove stands in Hong Kong as methodological testbeds, this study demonstrates that VR, coupled with 3D photogrammetry, can constitute a technically viable approach for representing physically demanding, environmentally sensitive, and access‐restricted ecosystems in an immersive digital environment. The resulting pilot multimodal platform delivers rich, interactive content spanning navigable virtual scenes, species hotspots, and multi‐format media assets, providing a functional proof‐of‐concept for this integrated methodology in complex natural terrains. Furthermore, by decoupling site exploration from geographical constraints, the online platform architecture demonstrates the technical capacity to serve a geographically distributed user base, offering a scalable framework for representing biodiversity‐rich environments such as Hong Kong's mangrove ecosystems. Users can navigate between different scenes, interact with species hotspots, and access information on various mangrove flora and fauna, including descriptions, photos, videos, and high‐resolution digital 3D models. Introductory videos on the ecology of the two sites and drone‐based aerial views were also incorporated to broaden the representational scope of the platform. While the virtual trail interface remains at a prototype stage, this pilot study primarily aims to establish the methodological feasibility of integrating VR and 3D photogrammetry into a multimodal digital platform, using mangroves as a model habitat. The platform is now positioned as a testbed for future empirical evaluation of its utility and effectiveness across ecologically critical yet less readily accessible environments.

Although some studies suggest that in‐person learning in field environments may still be more effective than virtual alternatives in environmental education (e.g., Wong et al. 2025), virtual learning tools can serve as valuable complements. They offer safer, more accessible, and often more cost‐effective experiential learning opportunities (Abdinejad et al. 2021; Cunnington et al. 2023). Furthermore, high‐resolution 3D models, by enabling multi‐angle visualization of key morphological identification features (Wang et al. 2022), may serve as a supplementary learning means of species identification alongside traditional photographic field guides. From a conservation standpoint, it is essential to strike a balance between environmental education and habitat conservation, especially in ecologically sensitive protected areas, such as the mangrove stands within the Ramsar Site featured in this study. This concern is increasingly relevant given the global rise in ecotourism and its potential impact on natural heritage sites (Zhang and Deng 2024).

### 4.2 Technological Novelty of the Digital Platform and Its Associated Inventory

Over the past few decades, VR has advanced rapidly. When integrated with other emerging technologies such as artificial intelligence (AI), its applications have expanded across diverse sectors, including education, agriculture, transportation, healthcare, teleconferencing, and tourism (Ribeiro de Oliveira et al. 2023). While the adoption of VR and 3D photogrammetry in education is gaining momentum (Kamińska et al. 2019; Chytas et al. 2024), their combined use in environmental education remains relatively underexplored. This study addresses that gap by leveraging recent advancements in photogrammetric techniques capable of producing high‐resolution digital 3D models of plant structures, detailed enough to support species identification (Wang et al. 2022). It further demonstrates that the technique may also allow for distinguishing intra‐specific morphological differences. This approach effectively overcomes a key limitation of traditional 2D photo guides, which struggle to convey the three‐dimensionality and fine detail necessary for students to learn key morphological traits for species identification.

Moreover, the platform, together with the voucher specimens stored in the Shiu‐Ying Hu Herbarium, introduces a robust and multi‐functional model for biodiversity inventory that integrates both digital data and physical materials. This hybrid approach may serve dual purposes: supporting scientific research and enhancing educational experience. The voucher specimens corresponding to the 3D models not only provide authoritative scientific references but also offer valuable hands‐on learning opportunities for students and visitors to the herbarium. In fact, 3D photogrammetry is emerging as an efficient and cost‐effective method for digitizing natural history specimens and other museum objects (Medina et al. 2020). The integration of various scientific components reflects the core philosophy and value of this study: combining validated scientific information with advanced technologies to create a comprehensive and dynamic inventory.

### 4.3 Limitations and Future Potentials

This study focuses primarily on the technical development of a platform that integrates VR and 3D photogrammetry. User experience and learning outcomes associated with the digital platform will require further evaluation, for example through the use of the questionnaires designed in this study and other systematic usability tests. In the short term, the VR‐compatible virtual trails will be made publicly accessible via the websites of the Shiu‐Ying Hu Herbarium (https://syhuherbarium.sls.cuhk.edu.hk) and the Hong Kong Biodiversity Information Hub (https://bih.gov.hk) to facilitate public use and engagement, as well as to collect feedback for improvement. The platform should therefore be understood as a technological prototype and research infrastructure that could support future investigation of its potential roles in environmental learning, and the communication of biodiversity information for sensitive natural sites.

Future development could incorporate additional mangrove sites and biodiversity datasets, as well as developing a more engaging and user‐friendly interface. Features such as AI‐guided tours may provide opportunities to examine how different forms of guidance affect usability, engagement, and learning. More generally, the study provides a basis for subsequent research into how digital technologies might contribute to nature appreciation and environmental education while reducing the need for physical access to sensitive habitats.

## 5 Conclusion

This study serves as a methodological case study that demonstrates the technical feasibility of integrating VR and 3D photogrammetry into a multimodal digital platform for the virtual exploration of Hong Kong's mangrove ecosystems. The platform responds to practical challenges of access and site sensitivity by providing navigable virtual scenes and specimen‐linked digital media. It also establishes a hybrid digital‐physical inventory that connects online assets with herbarium voucher specimens.

The study does not evaluate whether the virtual platform improves environmental learning, changes conservation‐related behavior, or contributes to conservation management. These outcomes should be treated as future research questions rather than demonstrated impacts. With continued technical development and empirical assessment, comparable platforms may have the potential to complement field‐based interpretation, broaden access to biodiversity information, and support communication about the value of sensitive ecosystems.

## Author Contributions

**Terence Pun Tung Ng:** conceptualization (equal), data curation (equal), formal analysis (equal), investigation (equal), methodology (equal), visualization (equal), writing – original draft (equal), writing – review and editing (equal). **Kenny Zhi Hao Li:** conceptualization (equal), data curation (equal), formal analysis (equal), investigation (equal), methodology (equal), visualization (equal), writing – original draft (equal), writing – review and editing (equal). **Cheryl Wood Yee Shum:** data curation (equal), formal analysis (equal), investigation (equal), methodology (equal), visualization (equal), writing – review and editing (equal). **Kian Ka Ki Tam:** data curation (equal), formal analysis (equal), investigation (equal), methodology (equal), visualization (equal), writing – review and editing (equal). **Yiu Man Chan:** data curation (equal), formal analysis (equal), investigation (equal), methodology (equal), visualization (equal), writing – review and editing (equal). **Flora Siu Yan Mok:** conceptualization (equal), methodology (equal), project administration (equal), supervision (equal), visualization (equal), writing – review and editing (equal). **David Tai Wai Lau:** conceptualization (equal), data curation (equal), formal analysis (equal), funding acquisition (lead), investigation (equal), methodology (equal), project administration (equal), supervision (equal), visualization (equal), writing – review and editing (equal).

## Funding

This project was funded by the Wu Jieh Yee Charitable Foundation to the Shiu‐Ying Hu Herbarium of The Chinese University of Hong Kong.

## Ethics Statement

The Agriculture, Fisheries and Conservation Department of the Government of the Hong Kong Special Administrative Region (HKSAR), which was involved in this project, is the local conservation authority responsible for authorizing access to the protected areas studied, including Mai Po Inner Deep Bay Ramsar Site. All sampling, photography, and videography, drone operations, and specimen preparation and storage were conducted in compliance with HKSAR legal requirements.

## Conflicts of Interest

The authors declare no conflicts of interest.

## Supporting information


**Data S1:** Data summary.

## Data Availability

The two virtual trails will be made publicly accessible through the websites of the Shiu‐Ying Hu Herbarium (https://syhuherbarium.sls.cuhk.edu.hk) and the Hong Kong Biodiversity Information Hub (https://bih.gov.hk). An Excel‐format summary file containing the key data of the paper and contact information for accessing the voucher specimens, raw 3D models/photos/videos and other relevant materials is availabe as Supporting Information.
